# The Journal’s Mission and Aims

**Published:** 1889-07

**Authors:** 


F. E. DANIEL, M. D., EDITOR AND PUBLISHER.

THIS JOURNAL, by unanimous vote of the members, was made the Official Organ of the Austin District Medical Society.

COLLABORATORS:

*Dr. R. M. Swearingen, Austin.*
*Prof. B. E. Hadra, M. D., Galveston.*
*Prof. Geo. Cupples, M. D., San Antonio.*
*Dr. T. C. Osborn, Cleburne, Texas.*
*Dr E. J. Doering, Chicago.*
*Dr. E. J. Beall, Fort Worth.*
*Dr Odo Betz, Germany.*
*Dr. J. W. McLaughlin, Austin.*
*Dr. T. J. Tyner, Austin.*
*Prof. J. F. Y. Paine, M. D., Galveston.*
*Dr. R. H. L. Bibb, Mexico.*
*Dr. T. J. Bennett. Austin.*
*Dr. Bat Smith, Wharton.*
*Dr. E. Meierhof, New York.*
*Prof. W. B. Rogers, M. D., Memphis, Tenn.*

### THE JOURNAL'S MISSION AND AIMS.

This issue of the JOURNAL begins the fifth volume. Four years of success have rolled away since it was launched upon the "uncertain sea," and that success has inspired confidence, and justifies the hope of a brilliant future. We begin the fifth volume with high hopes, and with our ambition to accomplish the high purposes for which it was begun, stimulated by the progress made in organization of the profession, as a means to further ends—the advancement, elevation and purification of the profession in Texas.

The chartering of the State Association, now organized upon a satisfactory basis, and built up to over five hundred members, largely "through the influence of spirited journalism" (*N. Y. Med. Record*) is the second step, and an important one, secured, we claim, mainly through the persistent advocacy of this JOURNAL. What the next step in the JOURNAL'S programme for the future will be can be gleaned from perusal of the excellent article on medical education, etc., from the *N. Y. Medical Record*, published herewith. Texas is overrun, as Illinois was, with the *refuse* of other States; it has been the common dumping ground of the United States, a kind of Botany Bay, to which fled and were driven, the imcompetents of other States, and the JOURNAL will cease not its efforts until the legislature is made to realize the true status, and induced to give us and the people, legislation that will work as wholesome a change in Texas as obtaine in Illinois.

Dr. Cupples, in his excellent report on "Medical Legislation," says: "O for a Rauch in Texas." It is not that: if Dr. Cupples and his committee had the same influence with, and backing by the legislature of Texas as Dr. Rauch has in Illinois, the Augean stable would have been cleaned out ere this.

To the accomplishment of regenerative and wholesome legislation. then, the JOURNAL addresses itself; and will know no stop nor stay until it is done; and for the promotion of medical science, and of professional brotherhood, it will exert its best talents.

